# The Transobturator Tape Versus Retropubic Tension-Free Vaginal Tape in the Treatment of Comorbid and Elderly Women With Stress Urinary Incontinence: A Retrospective Analysis of Over 800 Women

**DOI:** 10.7759/cureus.34072

**Published:** 2023-01-23

**Authors:** Sabine Schütze, Joana Behre, Benedikt Heitmeir, Juliane Schütze, Davut Dayan, Wolfgang Janni, Miriam Deniz

**Affiliations:** 1 Department of Obstetrics and Gynecology, University Hospital of Ulm, Ulm, DEU; 2 Applied Science, University of Applied Sciences, Jena, DEU

**Keywords:** complications, comorbidities, bmi, age, midurethral slings, stress urinary incontinence

## Abstract

Background and objective

The first-line surgical treatment for female stress urinary incontinence (SUI) involves midurethral slings (MUS), including the transobturator tape (TOT) and the retropubic tension-free vaginal tape (TVT). However, whether offering these procedures to older and comorbid women could lead to increased complications is a question that needs to be seriously addressed. In this retrospective cohort study, we aimed to compare the two procedures and evaluate the impact of age, BMI, and comorbidities on complications.

Materials and methods

A total of 873 procedures (306 TVTs/567 TOTs) performed between 2007 and 2017 were compared and correlated with regard to age, BMI, and comorbidities. Intraoperative complications included bleeding >50 ml, bladder injury, and anesthesia-associated complications. Postoperative complications included post-void residual volume, pain, hematoma, lower urinary tract infection, revision for loosening tape, and bladder infections. The comorbidities were evaluated based on the American Society of Anesthesiologists (ASA) and Charlson scores.

Results

A total of 873 MUS were conducted during the study period: 306 TVTs and 567 TOTs. Groupwise comparison between these procedures showed that women in the TOT group were older (p<0.001) with a higher BMI (p<0.001) and a higher ASA score (p<0.001) compared to the TVT group. Nevertheless, significantly more intraoperative complications, especially bladder injuries, were recorded in the TVT group. Postoperative complications occurred in 19.4% of the entire cohort, especially increased post-void residual volume. Postoperative hematoma and tape loosening were significantly more frequent in the TOT group. Age, BMI, and comorbidities showed no significant impact on intraoperative complications; however, the TOT procedure was associated with significantly fewer intraoperative complications [p=0.001, odds ratio (OR): 0.281].

Conclusions

Overall, both procedures were associated with a low number of perioperative complications. The TOT technique had a lower incidence of intraoperative complications. It must be highlighted that age and comorbidities had no influence on either the intra- or postoperative complication rates. Hence, we recommend that TOT is employed to treat SUI in older, more obese, and comorbid women.

## Introduction

Stress urinary incontinence (SUI) is a common disorder and is defined by the International Continence Society as “the complaint of any involuntary loss of urine on effort, physical exertion and/or on sneezing or coughing” [1]. The prevalence of this disorder among European women is estimated to be 35% [2]. Initial conservative treatment consists of pelvic floor muscle training, vaginal estrogen replacement, and lifestyle modifications [3]. Vaginal devices or oral medication such as duloxetine can also be utilized. Alternatively, there are several surgical procedures to correct this condition. Midurethral slings (MUS) are considered the gold standard modality for SUI in women; they have a good safety profile and have shown high efficacy in terms of short- as well as medium- and long-term outcomes [4]. MUS can be placed by the transoburator route [transobturator tape (TOT)] or the retropubic route [tension-free vaginal tape (TVT)]. The cure rates of both procedures are similar; however, the complication rates vary [3,5]. The retropubic approach is associated with higher rates of bladder perforation and voiding dysfunction, as well as vascular injuries and blood loss [4]. Groin pain is more frequently reported by patients who undergo the transobturator procedure. Vaginal erosion risk is low and comparable in both techniques [4]. Both procedures are minimally invasive and can be offered to older women and women with comorbidities. Several studies have shown favorable outcomes with these procedures but also reported an increased risk with advanced age with respect to the length of hospitalization, the risk of urinary tract infections, and de novo overactive bladder [6,7]. In light of this, this retrospective cohort study conducted at a tertiary care hospital aimed to compare TVT and TOT, analyzing the impact of age, BMI, and comorbidities on intra- and postoperative complications.

## Materials and methods

Ethical approval

The Ethics Committee of Ulm University approved this retrospective study (approval number: 380/18-FSt/se).

Study population

All patients treated with MUS between 2007 and 2017 were included in this study. The TVT group included 306 procedures while the TOT group included 567 procedures. For the TVT technique, the "Gynecare TVT Retropubic" was used, while the "Gynecare TVT Transobturator" was employed for the TOT technique. The inclusion criteria were as follows: patients with a diagnosis of SUI that required a surgical procedure, in whom previous conservative therapy options had been carried out but led to no improvement. There were no exclusion criteria.

Data collection

The following parameters were retrospectively collected: patient age and BMI, complications, the American Society of Anesthesiologists (ASA) score [8], and the Charlson score [9]. The ASA score is a physical status classification system applied before surgery with scores ranging from 1 (healthy) to 6 (brain death). The Charlson score estimates the morbidity and mortality of patients based on 19 prognostically relevant secondary diseases with scores assigned for each risk factor. Total scores are interpreted as follows, with regard to one-year mortality rate: 0: 12%, 1-2: 26%, 3-4: 52%, and >5: 85%. Complications were further classified into intraoperative complications (bleeding >50 ml, bladder injury, anesthesia-associated complications, and other complications), and postoperative complications (post-void residual volume ≥50 ml, pain, symptomatic hematoma, lower urinary tract infection, revision for loosening tape, bladder infection, and other complications). All these complications were detected in patients during their hospital stay. Every woman underwent vaginal sonography after the first micturition following the removal of the permanent catheter to determine the post-void residual volume. If this was too high, further controls were conducted since a loosening of the tape should be conducted in the early days. All women were tested for lower urinary tract infections. Each woman was examined by the surgeon postoperatively to diagnose a possible hematoma. Two certified pelvic organ prolapse surgeons undertook or supervised the procedures.

Statistical analysis

IBM SPSS Statistics V26 software (IBM Corp., Armonk, NY) for Windows was used for statistical analysis. Patient characteristics have been reported as mean, standard deviation (SD), and range for continuous data, and absolute and relative frequencies for categorized continuous and discrete data. Cross tabulations were performed to compare complication rates between the different treatment methods. For explorative interpretation, Chi-square tests were employed. To find predicting factors on complications, the logistic regression model was used.

## Results

Patients' baseline characteristics and groupwise comparison between TOT and TVT are presented in Table 1. The women in the TOT group were significantly older (p<0.001) and had a higher BMI (p<0.001) and ASA scores (p<0.001) (Table 1).

**Table 1:** Baseline characteristics of the entire cohort (n=873) with a groupwise comparison between the TOT and TVT groups *Statistically significant ASA: American Society of Anesthesiologists; TOT: transobturator tape; TVT: tension-free vaginal tape

Patient characteristics	TOT	TVT	P-value
Age in years			
Mean	60.25	54.63	<0.001*
Number of missing value	0	0	
Body mass index, kg/m^2^			
Mean	29.082	27.33	<0.001*
Number of missing values	0	1	
ASA score, n (%)			
1	83 (14.7%)	78 (25.6%)	<0.001*
2	260 (46.1%)	148 (48.5%)	
≥3	221 (39.2%)	79 (25.9%)	
Number of missing values	3	1	
Charlson score, n (%)			
≤1	515 (90.8%)	286 (94.4%)	0.064
>1	52 (9.2%)	17 (5.6%)	
Number of missing values	0	3	

Intraoperative complications

Intraoperative complications amounted to 3% in the entire cohort. Patients receiving TVT had significantly more complications than patients who underwent TOT (p=0.009). This was partially due to a significantly higher rate of bladder perforations in the TVT group (2.6%) compared to the TOT group (0.2%) (p=0.001). Blood loss >50 ml and anaesthesiologic and other complications were rare in both groups with no significant difference between the groups. Intraoperative complications for all patients and those for TVT and TOT separately are shown in Table 2.

**Table 2:** Comparison of intraoperative and postoperative complications between the groups *Statistically significant N=873: TVT: 306; TOT: 567 TOT: transobturator tape; TVT: tension-free vaginal tape

Intraoperative complications		P-value
Total intraoperative complications	26 (2.98%)	0.009*
TVT	17 (5.6%)	
TOT	9 (1.6%)	
Blood loss >50 ml	4 (0.46%)	0.615
TVT	2 (0.7%)	
TOT	2 (0.4%)	
Bladder injury	9 (1.03%)	0.001*
TVT	8 (2.6%)	
TOT	1 (0.2%)	
Anesthesiologic complications	6 (0.69%)	0.428
TVT	3 (1%)	
TOT	3 (0.5%)	
Other complications	8 (0.92%)	0.373
TVT	4 (1.3%)	
TOT	4 (0.7%)	
Postoperative complications		
Total postoperative complications	169 (19.4%)	0.221
TVT	66 (21.64%)	
TOT	103 (18.2%)	
Hematoma	8 (0.92%)	0.002*
TVT	7 (2.3%)	
TOT	1 (0.2%)	
Postoperative tape loosening	11 (1.26%)	0.046*
TVT	7 (2.3%)	
TOT	4 (0.7%)	
Postoperative pain	22 (2.54%)	0.879
TVT	8 (2.6%)	
TOT	14 (2.5%)	
Lower urinary tract infection	15 (1.72%)	0.414
TVT	7 (2.3%)	
TOT	8 (1.4%)	
Post-void residual volume	136 (13.3%)	0.266
TVT	51 (17.8%)	
TOT	77 (14.8%)	
Other complications	41 (4.7%)	0.059
TVT	20 (6.5%)	
TOT	21 (3.7%)	

Postoperative complications

A total of 19.4% postoperative complications were found. Increased post-void residual volume was the most frequent complication and occurred in 13.3% of all patients, more often in the TVT group (TVT: 17.8%; TOT: 14.8%) but with no significant difference between the groups. Of these patients, 1.3% needed revision surgery with secondary tape loosening. The tape had to be released significantly more often in the TVT group (2.3%) than in the TOT group (0.7%; p=0.046). The rate of postoperative hematoma was less than 1% but significantly higher in the TVT group (2.3% vs 0.2%; p=0.002). Postoperative pain was seen in 2.5% and lower urinary tract infections in 1.7% and this showed no difference between the groups. Postoperative complications for all patients and those for TVT and TOT separately are demonstrated in Table 2.

Factors influencing intra- and postoperative complications

A logistic regression was conducted using the factors of age, ASA score, Charlson score, BMI, and surgical procedure on intra- and postoperative complications (Table 3). The TOT procedure showed significantly fewer intraoperative complications [p=0.001, odds ratio (OR): 0.218]. Regarding postoperative complications, a higher BMI was associated with a significantly lower postoperative complication rate (p=0.014, OR: 0.790).

**Table 3:** Logistic regression analysis - factors influencing complications Boldface indicates statistical significance The factors analyzed were as follows: age; ASA classified into 1+2 and >3; and Charlson score classified into ≤1 and >1 ASA: American Society of Anesthesiologists; TOT: transobturator tape; TVT: tension-free vaginal tape

Variable	Intraoperative complications	Postoperative complications
Age	P=0.937	P=0.206
ASA score		
ASA 1+2	P=0.345	P=0.799
ASA 3	P=0.284	P=0.590
Charlson score		
≤1, >1	P=0.164	P=0.364
Body mass index	P=0.660	P=0.014, odds ratio: 0.790
Surgical procedure: TOT vs. TVT	P=0.001, odds ratio: 0.218	P=0.201

## Discussion

The objective of this single-center retrospective study was to compare TVT with TOT and evaluate the impact of age, BMI, and comorbidities on complications among a large sample of patients. The difference in baseline characteristics between the two cohorts has to be taken into account. The TOT procedure was offered to older women with a higher BMI and a higher ASA score. This could be attributed to the particular characteristics of the two surgical methods. The more difficult TVT procedure is preferable for patients with higher BMI, associated with a thicker abdominal wall. Due to the similar subjective cure rates of both procedures [10], the TOT is often the preferred modality. Even though the TOT group showed more demographic-related risks, the intraoperative complications were significantly lower. The main intraoperative complication, especially in the TVT procedure, was bladder injuries. This has been validated in the literature, based on the proximity to the bladder. In comparison to the Cochrane analysis by Ford et al. [4], fewer intraoperative bladder perforations were found in our cohort. This might be due to the high level of expertise of the surgeons who operated on our sample. Holdø et al. showed an increasing rate of bladder perforations and urinary retentions among patients whose TVT procedures were performed by "beginners" [11]. In contrast to their study, two certified pelvic organ prolapse surgeons undertook or supervised the procedures in this study. Barisiene et al. showed lower rates of bladder perforation (TVT: 1.79%; TOT: 0%) [12] compared to the findings in this study (TVT: 2.6%; TOT: 0.2%). However, our sample size was significantly larger (two-fold). Ford et al.'s study [4] showed a higher rate of bladder perforation in the TVT group (4.5%) compared to the TOT group (0.6%), which is in line with our findings.

It is well-known that urinary retention and voiding dysfunction are the most common complications following the MUS procedure [12,13], which is in line with the data presented in this study. The higher incidence of postoperative voiding dysfunction in our cohort compared to the literature [4,12] might be attributed to a standardized and precise postoperative control via transvaginal ultrasound. This enhanced the element of accuracy in our values. While the incidence of postoperative voiding dysfunction was higher than in the literature, only 1.3% of our patients required revision surgery with tape mobilization. This is lower compared to the studies of Barisiene et al. and Jahn et al., which showed rates of 2.4-3.5% [12,13]. Despite not reaching statistical significance, the TOT procedure showed lower postoperative voiding dysfunction. The rate of postoperative hematoma was low at 0.9%. However, significantly more patients in the TVT group had a postoperative hematoma compared to patients who had TOT, which is in line with Ford et al. [4] and might be due to the retropubic space, which is well-supplied by blood vessels.

Postoperative pain and lower urinary tract infection were not different between the two groups, which agrees with the outcomes of Elers et al. [14]. Only a few studies have looked into the correlation between comorbidities and perioperative complications within the MUS procedure. In this study, neither BMI nor ASA and Charlson scores of patients correlated with intraoperative complications. The TOT procedure was associated with significantly fewer intraoperative complications, which is in line with Barisiene et al. Their study observed surgery-related complications of 16.07% in the TVT group compared to 5.21% in the TOT group [12]. Regarding postoperative complications, only a higher BMI was associated with significantly fewer complications. Studies have offered conflicting findings in this regard so far. On the one hand, there are studies demonstrating that a higher BMI has no influence on the complication rate of MUS [15,16], while on the other, there are studies showing a rising complication rate with a higher BMI [13,17]. This finding can be attributed to the selection bias. More obese women received the TOT procedure, which is associated with a lower complication rate. All things considered, both procedures showed low intra- and postoperative complication rates. However, other studies have reported conflicting results. Some studies have described increased complication rates in elderly patients with a higher incidence of bladder perforations and a higher number of voiding difficulties after surgery [7,13,18-19]. However, the study by Stav et al. found comparable complication rates in younger and older women with no increased risk, especially for severe events [10]. Based on the findings of this study, the TOT procedure should be preferred for older, more obese, and comorbid women.

This study has a few limitations, primarily its retrospective design. In addition, there was no long-term follow-up of our cohort to evaluate known long-term complications including, but not limited to, vaginal erosion, de-novo urge, recurrent lower urinary tract infections, and persisting pain. Groin pain in particular is a known postoperative complication of the TOT procedure during the first six months [14]. Besides, a systematic survey of satisfaction rates among patients would have made our study more interesting. Furthermore, our research had a selection bias as predominantly older and more obese women were treated with TOT. However, even accounting for the mentioned bias, there were fewer intraoperative and postoperative complications in the TOT group, underlining the importance of individual treatment choices for every patient. A major strength of this study was its relatively large sample size of patients with SUI treated at a certified tertiary care hospital. Additionally, both TVT and TOT devices came from the same manufacturer, and no additional equipment was used. Moreover, during our study period (between 2007 and 2017), two certified pelvic organ prolapse surgeons undertook or supervised the procedures, and hence a high level of standardization of surgical intervention and postoperative care was ensured.

## Conclusions

Based on our findings, both TVT and TOT procedures for SUI therapy are generally associated with a low number of complications regardless of age or comorbidity. TOT procedure showed a favorable safety profile regarding intraoperative complications. It must be highlighted that age and comorbidities have no influence on intra- and postoperative complications. We believe our findings will prove valuable for surgeons when counseling elderly and comorbid patients with SUI.
